# Heterogeneous integration of contact-printed semiconductor nanowires for high-performance devices on large areas

**DOI:** 10.1038/s41378-018-0021-6

**Published:** 2018-08-13

**Authors:** Carlos García Núñez, Fengyuan Liu, William Taube Navaraj, Adamos Christou, Dhayalan Shakthivel, Ravinder Dahiya

**Affiliations:** Bendable Electronics and Sensing Technologies (BEST) Group, School of Engineering, University of Glasgow, G12 8QQ Glasgow, UK

## Abstract

In this work, we have developed a contact-printing system to efficiently transfer the bottom-up and top-down semiconductor nanowires (NWs), preserving their as-grown features with a good control over their electronic properties. In the close-loop configuration, the printing system is controlled with parameters such as contact pressure and sliding speed/stroke. Combined with the dry pre-treatment of the receiver substrate, the system prints electronic layers with high NW density (7 NWs/μm for bottom-up ZnO and 3 NWs/μm for top-down Si NWs), NW transfer yield and reproducibility. We observed compactly packed (~115 nm average diameters of NWs, with NW-to-NW spacing ~165 nm) and well-aligned NWs (90% with respect to the printing direction). We have theoretically and experimentally analysed the role of contact force on NW print dynamics to investigate the heterogeneous integration of ZnO and Si NWs over pre-selected areas. Moreover, the contact-printing system was used to fabricate ZnO and Si NW-based ultraviolet (UV) photodetectors (PDs) with Wheatstone bridge (WB) configuration on rigid and flexible substrates. The UV PDs based on the printed ensemble of NWs demonstrate high efficiency, a high photocurrent to dark current ratio (>10^4^) and reduced thermal variations as a result of inherent self-compensation of WB arrangement. Due to statistically lesser dimensional variations in the ensemble of NWs, the UV PDs made from them have exhibited uniform response.

## Nanosystems: Contact-Printing System Developed for Semiconductor Nanowires

A contact-printing system has been developed that allows the printing of semiconductor nanowires (NWs), such that electronic layers made from printed NWs can lead to devices with uniform response over large areas. With many attractive features, the NWs have gained attention in meeting demands to integrate low-power miniaturized devices over large areas and with flexible substrates. However, achieving response uniformity with nanoscale devices made from NWs has proved challenging. The team from Bendable Electronics and Sensing Technologies (BEST) group headed by Professor Ravinder Dahiya at the University of Glasgow, United Kingdom has developed a contact-printing system that overcome this challenge and efficiently prints NWs to various substrates while preserving the key characteristics and good control over their electronic properties. The technology has high reproducibility and reliability. The team believes its system holds promise for heterogenous integration of different semiconductor and metal NWs, allowing large-area scalability and and possibly the 3-D integration on flexible substrates.

## Introduction

Future electronics demands new ways for integration of low-power miniaturized devices over large areas and flexible substrates such as plastic, paper, fabrics, etc. In this regard, several approaches, novel materials and structures have been investigated and devices with improved performance, higher density, energy storage and sensitivity/selectivity have been obtained^1–5^. Among these, semiconductor nanowires (NWs) with attractive features related to quantum effects, higher surface sensitivity, higher thermal/electrical mobility, higher integrability and compatibility with flexible substrates are attractive for the development of high-performance photonics, photovoltaics, sensors, optoelectronics and electronics^6–8^. However, due to dimensional variability it is challenging to have response uniformity among the nanoscale devices made from NWs. The uniform device response is an important requirement for large-area electronics such as e-skin for robots^4^ and artificial neural network^5^. The smaller dimensions of NWs also increase the level of integration-related challenges especially for large-area electronics on non-conventional flexible substrates^3,9,10^. Considering these issues, new methods are needed to synthesize highly crystalline semiconductor NWs with uniform aspect ratios, and to assemble aligned NWs in a way that the electronic layers made from them could lead to devices having uniform response over large areas. Here, we present a contact-printing method to obtain such electronic layers from aligned NWs and to use the ensemble of NWs to develop devices. In contrast to single NW-based devices, the statistically dimensional variations are much lower in the case of ensemble of NWs, and therefore multi-NW-based devices have acceptable level of response uniformity over large areas.

A number of techniques have been investigated to transfer NWs from the growth to receiver substrate, with controlled location and print area, NW-to-NW spacing and NW surface/linear density, and preserving as-grown NWs properties (dimensions, morphology, crystal quality, etc.). Some of these methods such as transfer-printing^11–30^, dielectrophoresis^31–33^ and Langmuir–Blodgett^34–36^ have demonstrated high transfer yield, reproducibility, reliability and scalability towards large areas. Among these, transfer-printing techniques (e.g., contact-printing^13–26,30^, roll-printing^11^, combing^12,27,28^ and stamp-printing^29^) have demonstrated excellent potential for integration of semiconductor NWs on rigid and flexible substrates, exhibiting linear densities up to 10 NWs/μm^15^, and maximum surface coverage of 708 NWs/mm^2^ (Table 1)^17^. Furthermore, by assembling different kinds of NWs (doping type/level, bandgap energy, aspect ratios, etc.) at specific locations on receiver substrate field-effect transistors (FETs), high-electron mobility transistors, photodiodes, logic gates, active addressable photodetectors (PDs), etc. have been demonstrated (Table 1). However, the contact-printing mechanisms and operational parameters (contact force, sliding speed/stroke, conformal contact between donor/receiver substrates, etc.) are still not well investigated.

**Table. 1:** Comparison of various transfer-printing techniques for NW-based electronic devices

Assembly	Growth	Nanowire	NW average	Max. NW density	Key features	Ref.
technique	method	(NW)	diameter (nm)	(NWs/μm)		
Contact-printing	CVD	SWCNT	50	10	• Highest reported NW density • NWs on metal mesh for TEM	^15^
Roll-printing	CVD	Si	30	9	• NW transistor arrays on flexible substrates	^11^
Contact-printing	VLS	Ge	30	8	• Wafer-scale assembly of bottom-up NWs	^23^
Contact-printing	VLS	ZnO	100	7	• NWs heterogeneous integration • WB UV PD	This work
	MACE	Si	115	3		
Contact-printing	VLS	SnO2	80–120	6	• NW thin film transistors • Max. 0.3 NWs per μm^2^	^22^
Contact-printing	VLS	Ge/Si	30	5	• NW active matrix e-skin	^20^
Contact-printing	VLS	Ge/Si core/shell	12	4	• Printing of 1–10 layers of multi-NWs for flexible FET	^24^
Contact-printing	PVT	InAs	30	4	• NW transistors operating in GHz frequency range	^21^
Contact-printing	CVD	CdSe Ge/Si	30 12	3–4	• Heterogeneous integration • NWs PD circuitry with image-sensing functionality	^14^
Contact-printing	CVD	Ge Si	30	2–5	• Contact-printing on functionalized substrates	^19^
Combing	VLS	Ge InAs	60 30	2.2	• Polymer-based combing to fabricate NW FET	^12^
Contact-printing	CVD	InAs	20–40	2	• High-mobility NW transistors	^18^
Combing	VLS	Si Ge/Si	50	1.5	• 98.5 ± 1% directional alignment	^28^
Contact-printing	CVD	Zn3P2	50–200	1.3	• Rigid/flexible PD	^12^
Combing	VLS	Ge/Si Core/shell	15	1.1	• 60% Single NW devices • 22% double NW devices	^27^
Contact-printing	CVT	Zn2GeO4	150	0.6–1	• High voltage stability FET • High performance PD	^13^
		In2Ge2O7	100–125			
Contact-printing	CVD	ZnO	450	0.1–0.2	• Printing of NWs on PDMS	^25^
Contact-printing	CVD	ZnO	200	0.5	• Max. 40 NWs per mm^2^	^26^
Contact-printing	CVD	CdSxSe1-x	–	–	• Array of PDs	^16^
Contact-printing	CVD	Si	50–250	–	• Max. 708 NWs per mm^2^	^17^
	Solution	Si	290			
Stamp-printing	VLS	SWNTs	–	–	• Printing of Si, GaAs, and GaN NWs and SWNTs	^29^
	VLS	GaAs	270			
	VLS	GaN	–			

*CVD* chemical vapour deposition, *VLS* vapour–liquid–solid, *MACE* metal-assisted chemical etching, *PVT* physical vapour transport, *CVT* chemical vapour transport

This work presents a detailed description of a home-made contact-printing system with close-loop control. The system has high transfer yield for both bottom-up and top-down semiconductor NWs from the growth substrate to defined location on the receiver substrate. The high reliability and reproducibility achieved by the developed system allows an accurate control over the NW printing process through operational parameters (e.g., contact pressure between donor (NW substrate) and receiver (foreign substrate) substrates and the sliding speed/stroke of the receiver substrate). The NW transfer yield, evaluated from resulting NW density (NWs/μm) and NW-to-NW spacing, has been analysed as a function of the above printing parameters. Finally, contact-printing method has been successfully used to fabricate ultraviolet (UV) photodetectors (PDs) with ZnO and Si NWs multi-NW electronic layers—printed in a Wheatstone bridge (WB) configuration—acting as the photosensitive material and electronic component, respectively. The compatibility of the developed method with non-conventional substrates was demonstrated by the successful fabrication of WB UV PDs on rigid and flexible substrates. The reliability and thermal stability of multi-NW UV PDs in WB configuration is compared to single NW-based UV PDs.

## Results

### Contact-printing system

**Fig. 1:**
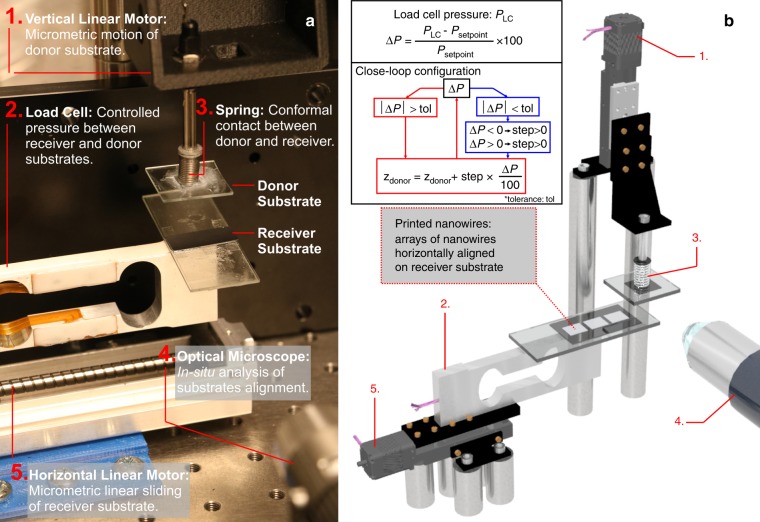
Description of Contact-printing Setup. **a** Image and **b** schematic illustration of the contact-printing system. A linear stage motor allows a micrometric movement control of the donor substrate’s vertical position with respect to the receiver substrate (1). The force exerted by the donor substrate on the receiver substrate is measured by a load cell placed underneath (2). The spring attached to the donor substrate ensures its conformal contact with receiver substrate (3) and the alignment is checked by in-situ analysis by optical microscopy (4). The system also allows controlling the sliding speed/stroke of the contact-printing by using a second linear stage motor (5). Inset: contact-printing experiments are carried out at a specific pressure controlled by a close-loop configuration described in the logic diagram

The contact-printing system developed in this work is presented in Fig. 1. The system consists of: (1) a vertical linear position motor to control the position of the donor substrate, (2) a load cell to measure the force exerted by the donor substrate when they come in contact with the receiver substrate, (3) a three-dimensional (3D) printed platform with a spring to ensure the conformal contact between donor and receiver substrate (Fig. 1, also see Supplementary Movie 1), (4) an optical microscope to analyse the alignment and conformal contact between donor and receiver substrates (see Fig. S1) and (5) a horizontal linear position motor to control the sliding movement of the receiver substrate during the contact-printing process. Accordingly, the contact force (*F*) exerted on the receiver substrate is measured as a function of the donor vertical displacement (*z*), resulting in a linear tendency given by *F*(N) = 0.55 + 8.7 *z*(mm) (see FIG. S2(a)). In addition, we have determined the sensitivity (*S*) of the load cell as a function of the motor step size (see FIG. S2(b)), aiming to determine the minimum step size that we can use to produce an appreciable variation in the force measured by the load cell. From this study, we have concluded that *S* of the load cell is around 5% for steps of 5 μm. Accordingly, contact-printing experiments carried out in this work uses a minimum step size of 5 μm, allowing an accurate measurement over the applied force range.

Once the location of NW transfer and the set-point pressure (*P*_set-point_) are entered into the system, the close-loop configuration (Fig. 1b), with control parameters such as the approach step size and the tolerance (tol), allows it to reach *P*_set-point_ within a short period of time (<1 min). A Labview interface has been developed to guide the user at each step of the contact-printing process (see FIG. S3). Firstly, parameters such as donor substrate area, contact pressure, sliding speed/stroke, vertical motor step size and tolerance are defined in the programme. Thereafter, both donor and receiver substrates are loaded in the system. The vertical motor moves the donor substrate towards the receiver substrate surface. Once the load cell detects the formation of contact between the donor and receiver substrates, the pressure measured at each step of the vertical motor (*P*_LC_) is compared to *P*_set-point_. For *P*_LC_ < *P*_set-point_, the vertical motor continues to move towards receiver, i.e., step × Δ*P*/100 < 0, and the step length is reduced in proportion to the difference between pressures (Δ*P* = 100 (*P*_LC_  − *P*_set-point_)/*P*_set-point_), i.e., step_*i*+1_ = step_*i*_ × Δ*P* (Δ*P* < 0 implies forward direction). For *P*_LC_ > *P*_set-point_, the vertical motor moves the donor substrate away from the receiver. Once the tolerance is reached, i.e. |Δ*P*| *≤* tol, the receiver substrate slides at a speed (*v*_sliding_) for a stroke (*l*_sliding_) programmed in the horizontal motor. The latter also allows control over the surface to be covered by NWs as per the circuit layout, which is demonstrated later. For the sake of clarity, a recording of the above process is provided in the Supplementary Movie 2.

### Nanowire synthesis

The accurate synthesis of NWs is a key aspect of the fabrication of devices based on such long aspect ratio nanostructures. In this work, we have thoroughly investigated bottom-up and top-down approaches to obtain ZnO and Si NWs, respectively, with high crystal quality and vertically aligned on the growth sapphire. During this investigation, we have focused on the analysis of the growth parameters to ensure a high degree of alignment and uniform dimensions of the resulting NWs, which will greatly benefit the contact-printing process performance, i.e., improvement of the NW alignment, increase of the density of printed NWs, reduction of the NW-to-NW spacing and preservation of the initial NW length, as it will be shown later on.

#### Bottom-up nanowires

**Fig. 2:**
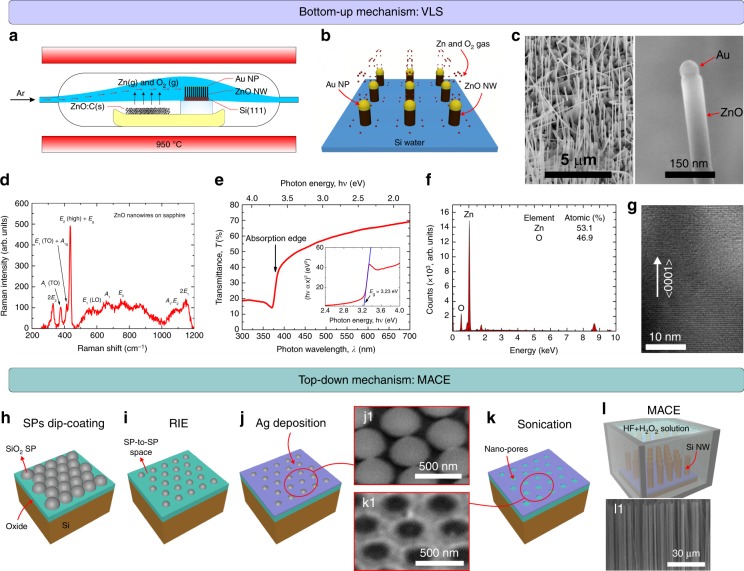
Synthesis and Characterization of ZnO and Si Nanowires. **a**–**g** Bottom-up ZnO NWs grown by CVT. **a** 2D schematic illustration of CVT process. **b** 3D schematic illustration detailing VLS growth mechanism of ZnO NWs using Au NPs as catalyst. **c** SEM images of ZnO NWs vertically aligned on Si(111) substrate. Characterization of ZnO NWs by **d** Raman spectroscopy, **e** transmission spectrophotometry, **f** EDX, and **g** TEM. Inset of (**e**) shows Tauc’s plot for *E*_g_ analysis. **h**–**l** Top-down Si NWs grown by MACE. **h**–**k** 3D schematic illustration of MACE process, comprising (**h**) large-area dip-coating of SiO_2_ SPs on Si(100) substrate, **i** SPs size reduction by RIE, **j** Ag deposition by thermal evaporation (j1: SEM image), **k** SP removal by sonication (k1: SEM image), and **l** MACE synthesis (l1: SEM image)

ZnO NWs are synthesized on Si(111) substrates by chemical vapour transport (CVT) technique in a quartz tube placed inside a high temperature horizontal furnace (Fig. 2a). The bottom-up growth mechanism of NWs is based on the well-established vapour–liquid–solid (VLS) mechanism (Fig. 2b)^37^. In VLS, Au nanoparticles (NPs) are typically used as catalyst, acting as liquid traps for Zn and O_2_ gas species. In this work, a 4 nm thick Au layer has been evaporated on top of a Si(111) substrate and annealed at 1050 °C for 10 min in Ar ambient, resulting in a random distribution of Au NPs (see FIG. S4). In the system geometry used in this work (Fig. 2a), a high Ar flow rate of 1000 sccm is observed to favour the growth of ZnO NWs vertically aligned on the Si(111) substrate (Fig. 2c). In contrast, a lower Ar flow rate is observed to hinder the NW length uniformity along the substrate surface (see FIG. S5) or even prevent ZnO nucleation with the shape of a NW (see FIG. S6)^38,39^. In this scenario, Au NPs become saturated and remain atop NWs tip (Fig. 2c), which is critical for the VLS growth continuity. As observed by scanning electron microscopy (SEM), the CVT growth of ZnO NWs for 1 h results in NWs with an average length and diameter of 10 μm and 100 nm, respectively. Raman spectroscopy confirms the high crystal quality of NWs (Fig. 2d). For optical characterization, ZnO NWs have been transferred from growth substrate to an ethanol solution by sonication. Figure 2e shows transmittance of NWs solution measured by ultraviolet/visible spectrophotometry (UV2600 Shimadzu) at wavelengths (*λ*) ranged between 300 and 700 nm. Transmittance spectrum shows an absorption edge at *λ ∼* 380 nm, exhibiting a direct wide band gap energy (*E*_g_) of around 3.23 eV, as confirmed by Tauc’s Plot (see inset of Fig. 2e), demonstrating the well-known sensitivity of ZnO NWs to UV light range^33^. Energy dispersive X-ray diffraction (EDX) of ZnO NWs shows a highly stoichiometric structure (Zn: 53.1%, O: 46.9%), without any trace of contaminants (Fig. 2f). Transmission electron microscopy (TEM) also confirms the high crystalline structure of resulting NWs, demonstrating that NWs follow *c*-axial direction (Fig. 2g).

#### Top-down nanowires

Si NWs have been synthesized through the top-down approach namely metal-assisted chemical etching (MACE). In this work, SiO_2_ spheres (SPs) were dip-coated on the Si substrate surface Fig. 2(j1), forming a self-assembled monolayer (SAM) that was used as a mask to create a nano-porous metallic layer Fig. 2(k1). In MACE, the areas under the metal are etched away, resulting in vertically aligned Si NWs on the Si wafer as observed by SEM (Fig. 2(l1)). In this regard, the length of Si NWs can be controlled by the MACE time, with an etching rate of 1.33 μm/min. We have experimentally probed the MACE synthesis of Si NWs with lengths up to 100 μm. For this work, we will use Si NWs with an average length of 10 μm.

### Analysis of contact-printing performance

#### Contact-printing mechanism

**Fig. 3:**
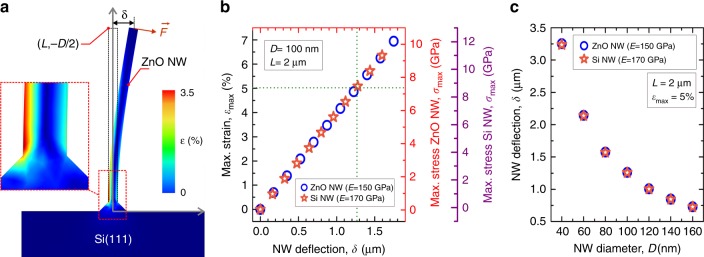
Simulation of Contact-printing Mechanism. **a** 2D simulation of the ε produced along a single ZnO NW body subjected to a force (*F*) normal to the NW surface. The loading point is considered at the top part of a NW with length (*L*) of 2 μm and diameter (*D*) of 100 nm. The NW deflection (*δ*) is measured between the NW edge and the neutral plane. Inset: higher magnification image of the NW root, presenting the *ε*_max_ along the NW stem. **b** *ε*_max_ and *σ*_max_ vs. *δ*, for both Si (*E* = 170 GPa)^40^ and ZnO NWs (*E* = 150 GPa)^41^, where *ε*_max_ and *σ*_max_ have been calculated from expressions (3) and (4), respectively. **c** *δ* vs. *D* considering both Si and ZnO NWs and assuming an average fracture strain of 5%

Prior to the experimental transfer of ZnO and Si NWs using the contact-printing system shown in Fig. 1, we have analysed the printing mechanism of NWs as a function of the NW aspect ratio, NW material and applied contact pressure. This study aims to find the range of contact pressures that would lead to reach the fracture limit of a single NW, preserving its maximum original length, and to understand the breaking mechanism of a NW as a function of its dimensions. In this regard, we have used COMSOL Multiphysics for two-dimensional (2D) finite elements (FE) simulation of a single NW vertically aligned on a Si substrate, and the response of that NW to different bending conditions, including: (1) the analysis of the maximum strain (*ε*_max_) and maximum stress (*σ*_max_) regions along the NW body (Fig. 3a), (2) the dependence of *ε*_max_ and *σ*_max_ with respect to the NW deflection (*δ*) (Fig. 3b) and (3) the dependence of *δ* with respect to the NW diameter (*D*) (Fig. 3c).

To evaluate the *ε*_max_ within a single NW in terms of the fracture strain and the elastic modulus (*E*), we have simulated the bending of a NW by subjecting it to a follower force (***F***), i.e., a force that is applied at the loading point (*L*,-*D*/2) and is always normal to the NW side surface (Fig. 3a). Results of the FE simulation show that *ε*_max_ and *σ*_max_ are close to the NW root, which means during the contact-printing process the fracture of the NW is likely to initiate in that region and at the side surface subjected to a tensile stress (see inset of Fig. 3a).

In addition to FE simulation, for a uniform NW subjected to a bending conditions as observed during a contact-printing process, the *ε*_max_ and *σ*_max_ can be calculated through the beam theory^41^

1 $$\varepsilon _{\max } = \frac{3}{2}\frac{{D\delta }}{{L^2}}$$

2 $$\sigma _{\max } = E\varepsilon_{\max } = \frac{3}{2}\frac{{D\delta }}{{L^2}}E,$$

where *D* is the NW diameter, *δ* is the deflection measured at the loading point (*L*,-*D*/2) (Fig. 3a) and *E* is the Young’s modulus of the material^40,41^. Analysing the *ε*_max_ and *σ*_max_ as a function of *δ* (Fig. 3b), one can observe that both ZnO and Si NWs present the same linear trend. Assuming an average fracture strain of 5 ± 2%, as experimentally determined for ZnO^41^ and Si NWs^3^, from Fig. 3b one can conclude that the fracture of ZnO and Si NWs with a *L*/*D* of around 20 occurs for *δ* above 1.25 μm. Moreover, the fracture of ZnO and Si NWs comprises a *σ*_max_ of around 7.3 GPa and 8.9 GPa, respectively, which are around 3 orders of magnitude higher than those obtained for bulk ZnO (~MPa) but is similar to those obtained for bulk Si^41^. Moreover, we have analysed the effect of *D* on the *δ* required to fracture a NW for a constant *L* of 2 μm (Fig. 3c). From this analysis, it can be noticed that the *δ* leading to the fracture of the NW decays exponentially with *D*.

The above results indicate that the contact-printing mechanism requires a continuous and progressive bending of the NWs to reach the fracture strain close to the root of the NW. This can be achieved by using both a constant contact pressure between the donor and receiver substrates and a micrometric sliding stroke and this is the reason for using spring mechanism in the proposed experimental arrangement. However, the *δ* observed under fracture conditions is strongly dependent on the NW diameter as depicted in Fig. 3c, where the *δ* increases with *D*. Since our donor sample consists of vertically aligned NWs with a narrow distribution of diameters along the substrate surface (Fig. 2c, l1), one can understand short range of *δ* (>1.25 μm) will be needed during the same contact-printing experiment to ensure a high NW transfer yield.

#### Nanowire transfer analysis

**Fig. 4:**
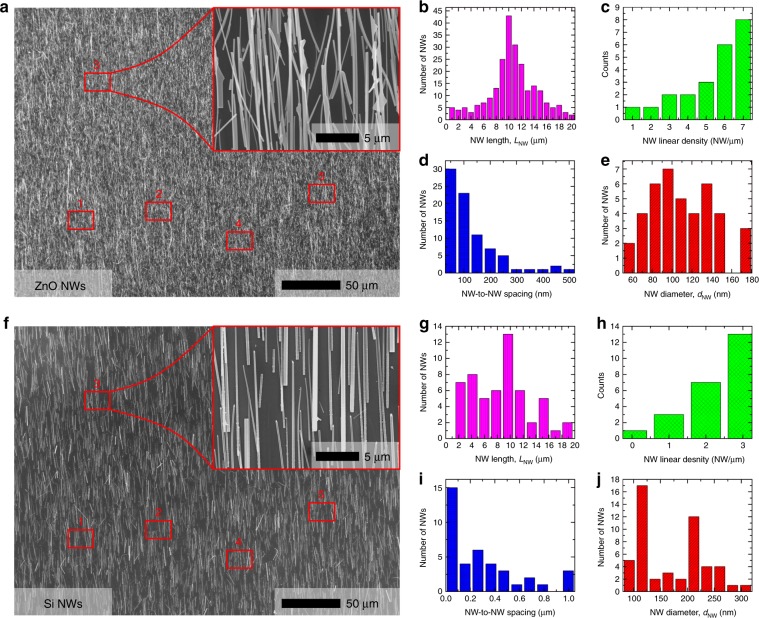
Method to Analyse Contact-printing Performance. Optical microscope and SEM images (insets) of (**a**) ZnO and **f** Si NWs contact-printed on Si(100) substrates. Statistical analysis of contact-printing performance (**b**–**e**) for ZnO and **g**–**j** for Si NWs, obtained from 5 randomly chosen areas in the total sample area of 1 × 1 cm^2^ to analyse (**b**, **g**) NW length, **c**, **h** NW density, **d**, **i** NW-to-NW spacing and **e**, **j** NW diameter, along a 10 μm long horizontal profile (insets of **a**, **f**)

We have studied the transfer performance of our system by characterizing the morphology of as-printed NWs by means of optical microscopy and SEM and calculating figure of merits such as NW length after the printing, NW density (NWs/μm) and NW-to-NW spacing. The dimensions of the donor or growth substrate used in this experiment are around 1 × 1 cm^2^. We have carried out a statistical analysis of different areas for both ZnO NWs (Fig. 4a-e) and Si NWs (Fig. 4f-j). As an example, Fig. 4a, f shows the ZnO and Si NWs, respectively, contact-printed on a Si(100) receiver substrate using a force of 5 N (50 kPa) and a sliding speed of 100 μm/s. For the statistical analysis of the contact-printing performance, up to 5 different areas (insets of Fig. 4a, f) have been randomly chosen in the total area of 1 × 1 cm^2^. The distribution of NW size (diameter and length), NW-to-NW spacing and NW density (NWs/μm) are calculated along a 10 μm length horizontal profile drawn in the SEM figure (see insets of Fig. 4a, f). From this analysis, one can extract the following information: (1) the average length of contact-printed ZnO and Si NWs is around 10 μm (Fig. 4b, g) which is similar to the as-grown NW length obtained from VLS and MACE synthesis and is also in agreement with COMSOL simulation (Fig. 3); (2) the maximum NW density is around 7 NWs/μm for ZnO NWs (Fig. 4c) and 3 NWs/μm for Si NWs (Fig. 4h); (3) the average NW-to-NW spacing is about 165 nm in ZnO and 455 nm in Si NWs (Fig. 4d, i); and (4) the average diameters of ZnO and Si NWs are around 95 (Fig. 4e) and 115 nm (Fig. 4j), respectively.

## Discussion

### Contact-printing performance: role of the contact pressure

Contact-printing of ZnO and Si NWs donor substrates (dimensions of 1 × 1 cm^2^) has been carried out using the system described in Fig. 1, under different conditions comprising a contact force (*F*) between 1 and 5 N, and a constant *v*_sliding_ and *l*_sliding_ of 100 μm/s and 1 mm, respectively. In this section, we analyse the effect of *F*, and its equivalent contact pressure, on parameters such as NW density and NW-to-NW spacing obtained right after the contact-printing of above NWs on Si(100) substrates. This study aims to determine the optimum conditions allowed by our system for high-performance contact-printing of uniform NW-based electronic layers over large areas, and analyse the outcome by printing NWs grown by bottom-up and top-down approaches (Fig. 2).

**Fig. 5:**
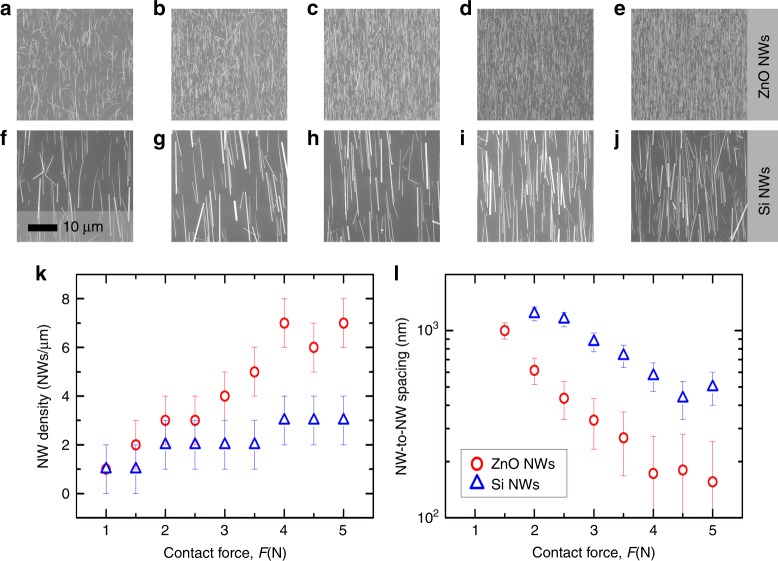
Effect of Contact-printing Force on Nanowire Printing Dynamics. SEM images of (**a**–**e**) ZnO and **f–j** Si NWs contact-printed on Si(100) substrates using (**a**, **f**) 1 N, (**b**, **g**) 2 N, (**c**, **h**) 3 N, (**d**, **i**) 4 N and **e**, **j** 5 N, and a constant *v*_sliding_ of 100 μm/s along a *l*_sliding_ of 1 mm. **k** NW density and **l** NW-to-NW spacing obtained from SEM images and represented as a function of 1 N < *F* < 5 N (steps of 0.5 N) for ZnO and Si NWs

Figure 5 shows the SEM images of contact-printing outcome with ZnO NWs (Fig. 5a–e) and Si NWs (Fig. 5f–j) at different *F*, comprising (5a, f) 1 N, (5b, g) 2 N, (5c, h) 3 N, (5d, i) 4 N, and (5e, j) 5 N. From the SEM images, one can conclude that: (1) both kinds of NWs are successfully contact-printed for the specific range of forces analysed here; (2) for *F* > 1.5 N in the case ZnO and *F* > 2 N for Si, the transferred NWs are highly aligned along the sliding direction (>90%); (3) NW density increases with *F*, showing highest values of 7 and 3 NW/μm for ZnO and Si NWs, respectively (Fig. 5k); and (4) the NW-to-NW spacing decreases with *F*, exhibiting a minimum average value of 165 and 455 nm for ZnO and Si NWs, respectively (Fig. 5l). The observed NW densities are close to those reported in the literature for contact-printed semiconductor NWs, e.g., Ge NWs (8 NWs/μm)^23^, Si NWs (9 NWs/μm)^11^ and carbon nanotubes (CNTs) (10 NWs/μm)^15^, and to the best of our knowledge, is the highest reported for ZnO NWs using contact-printing technique. Moreover, the high NW density has been demonstrated over large areas up to around 1 × 1 cm^2^, showing the potential scalability of the approach (see FIG. S7). Error bars included in Fig. 5k, l represent the statistical variation obtained from 5 different areas analysed along the 1 × 1 cm^2^ total sample area. This variation is lower for higher densities of printed NWs, e.g., at 5 and 7 NWs/μm the variation is estimated around 20% and 14.28%, respectively. The variation over such large areas depends on several factors, including contact-printing operational parameters and substrates properties such as smoothness or thickness variation of the receiver substrate. In this work, we have assumed that the receiving substrate is smooth with uniform thickness over the transfer area.

The successful transfer-printing of both ZnO and Si NWs over large areas is possible due to accurate control of our system, and the soft and conformal contact formed between donor and receiver substrates which preserves the NW length and prevents structural damage during the contact-printing process. This unique feature of our system allows us to print electronic layers from different kinds of semiconductor NWs on pre-defined regions ranging from few mm^2^ to tens of cm^2^.

### UV photodetector fabricated by contact-printing

Typically, NW PDs based on light-dependent resistance mechanism^42^ use a voltage divider circuit to measure the voltage difference across the load resistance when the PD is exposed to different lights^33^. In this regard, WB circuits offer greater advantages than a simple voltage divider and these include higher sensitivity and self-compensation of external effects such as temperature, humidity, vibrations, etc^43,44^. Here, we have demonstrated the potential of contact-printing technique to integrate different kinds of semiconductor NWs, and densities of NWs to fabricate a WB circuit fully based on NW electronic layers. Accordingly, we show the step-by-step fabrication procedure of a UV PD based on a WB by using contact-printing and then the characterization of the resulting PD in dark and under UV illumination.

#### UV photodetector fabrication steps

**Fig. 6:**
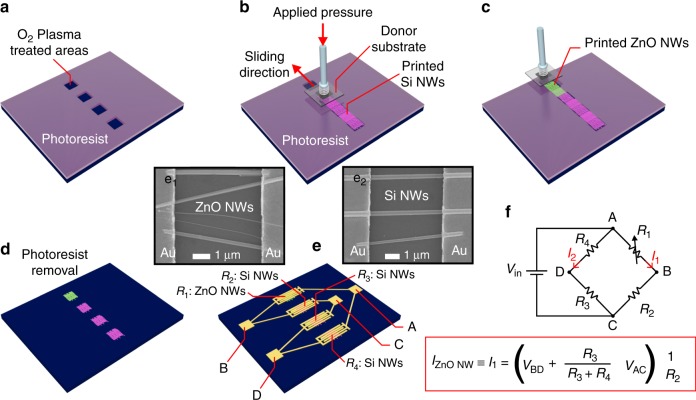
3D Schema of UV Photodetector Fabrication. **a–e** Fabrication steps of UV PDs based on ZnO and Si NWs, comprising: **a** definition of 20 mm^2^ areas on a S1818 photoresist layer by photolithography, followed by an O_2_ plasma treatment (100 Watt and 0.3 mbar for 1 min); contact-printing of (**b**) Si and **c** ZnO NWs; **d** removal of the photoresist in warm acetone (50 °C for 2 min); **e** definition of Ti(4 nm)/Au(200 nm) interdigitated electrodes by photolithography and lift-off, where (e_1_) and (e_2_) show SEM images of printed ZnO and Si NWs, respectively, bridging a pair of Ti/Au electrodes with a 5 μm gap. **f** WB equivalent circuit and the expression determining the electric current flowing through ZnO NWs (*I*_ZnO NW_)

Figure 6a–e shows a 3D schematic illustration of the step-by-step experimental procedure used in this work to fabricate UV PDs based on WB configuration. A Si(100) substrate with a 300 nm thick layer of SiO_2_ on top was used as a receiver substrate. Firstly, four NW assembling areas (2 × 10 mm^2^) were defined with S1818-positive photoresist by photolithography as shown in Fig. 6a. Prior to the contact-printing process, the receiver substrate was exposed to an O_2_ plasma (total pressure 0.3 mbar, 40 sccm of O_2_ flux and 100 Watt) for 1 min using an Oxygen Barrel Asher (PlasmaFab 505) to promote the hydroxylation of the Si surface, i.e., the formation of –OH groups covering the open areas (Fig. 6a). The role of the chemical properties of the receiver substrate surface on the density of contact-printed NWs has been rarely reported in the literature^23^. Essentially, during the contact-printing process, the stickiness of the receiver substrate surface is required to enhance the adhesion of NWs to the receiver substrate. It is well known that hydrophobic and hydrophilic surfaces result in non-sticky and sticky surfaces, respectively. Surface functionalization^23^ and the hydroxylation in O_2_ plasma environments (the latter reported in this work), have demonstrated highly hydrophilic surfaces in Si/SiO_2_ substrates. In this regard, the strong interaction required between the receiver substrate and the NWs could be fostered by using hydrophobic receiver substrates. This is expected to eventually promote the detachment of the NWs from the growth substrate, and their subsequent transfer to the receiver substrate. In these conditions, the maximum compactness of the printed NW-based layer is limited by the dangling bonds characteristic of each SAM or coating. Another potentially promising approach for the improvement of the density of printed NWs is the development of contact-printing of NWs on so-called super-hydrophilic-coated substrates^45^.

Right after the receiver substrate pre-treatment, Si NWs were contact-printed continuously on the receiver substrate with the photoresist, covering three out of four defined areas (Fig. 6b). Based on the geometry and dimensions of the electrodes, the total area covered by Si NWs is around 6 × 10 mm^2^. Therefore, the dimension of the donor substrate (Fig. 2(l1)) is slightly bigger than the electrodes area (7 × 10 mm^2^). Using the linear positioning stages of the contact-printing system (Fig. 1), the donor substrate is aligned at the specific region where Si NWs is to be printed. Then, both donor and receiver substrates are brought in contact under a force of 5 N, followed by the sliding of the donor along a stroke of 1 mm at a speed of 100 μm/s. In the same way, a ZnO NW-based donor substrate with dimensions of around 3 × 10 mm^2^ is contact-printed on the remaining area (2 × 10 mm^2^) using a sliding speed of 100 μm/s and a force of 2.1 N, resulting in the same pressure of around 70 kPa (Fig. 6c).

Sample morphology was re-analysed by SEM to evaluate the outcome of contact-printing on top of patterned photoresist with respect to the situation when the process is directly carried out on Si(100) substrates (Fig. 4). Results of this analysis show similar assembling performance like those shown in Fig. 4, i.e., NW linear densities around 5–6 and 2–3 NWs/μm for ZnO and Si NWs, respectively. However, after the photoresist is softly removed in warm acetone (50 °C) for 2 min, and the organic leftovers cleaned in isopropano for 2 min (Fig. 6d), the average NW linear densities of ZnO and Si NWs in the patterned area decrease down to 1 and 0.5 NW/μm, respectively, mainly due to the unintentional removal of NWs during the solvent cleaning. The hydroxylated surface of the receiver substrate has demonstrated to have a strong effect during the contact-printing process but has shown a poor adhesion between substrate and aligned NWs during the post-processing of the device. In this regard, wet pre-treatments of receiver substrates and the functionalization processes^23^ have been demonstrated to improve the adhesion between NWs and substrate. These could be alternative options for preserving the NW density after several post-processing steps, and would allow a monolithic fabrication of NW-based 3D devices^24,29^.

Finally, four arrays of metallic interdigitated electrodes Ti(4 nm)/Au(200 nm) with a gap length and width of 5 μm and 10 mm, respectively, and a total number of 12 gaps per array, are deposited by e-beam evaporation technique and defined by photolithography and lift-off (Fig. 6e). The linear geometry of the interdigitated electrodes presented in Fig. 6e has been designed to favour the integration of NWs using only two steps contact-printing, one for each type of NWs. SEM characterization after the definition of electrodes shows that ZnO (Fig. 6(e_1_)) and Si (Fig. 6(e_2_)) NW density is preserved (i.e., ~1 ZnO NW/μm and ~0.5 Si NW/μm) which demonstrates the robustness and stability of the bridge formed between electrodes. In spite of the NW density reduction after the photoresist removal, the fabrication of multiple devices following the process described in Fig. 6 has demonstrated similar PD response as shown below. The connections in Fig. 6e are schematically described in the equivalent circuit of Fig. 6f, where ZnO NWs act as *R*_1_, and Si NWs act as *R*_2_, *R*_3_, and *R*_4_ in a WB configuration. *R*_1_ to *R*_4_ have been measured individually (i.e., cutting the device into four pieces and electrically insulating each resistance from others), resulting in *R*_1_ of 571 ± 50, *R*_2_ of 16 ± 8, *R*_3_ of 10 ± 3 and *R*_4_ of 11 ± 3, with the error calculated from the results obtained from 3 different WB devices fabricated by contact-printing. Based on WB expressions (see FIG. S8), resistances obtained from aforementioned contact-printing process result in an unbalanced WB, i.e., *V*_out_ ≡ *V*_D_ *−* *V*_B_ ≠ 0. Typically, balanced WB configuration is used in sensing applications mainly due to the high sensitivity of *V*_out_ to external factors such as light. Following the procedure described in Fig. 6a–e, we have increased *R*_2_ from 16 Ω up to 572 Ω by reducing the contact-printing area of Si NWs from 2 × 10 mm^2^ down to 2 × 5 mm^2^ in only one of the electrodes arrays. In this scenario, the initial *V*_out_ measured in dark conditions is around 400 μV, which is considered a balanced WB state (*R*_1_/*R*_2_ ~ *R*_4_/*R*_3,_ see FIG. S8).

Figure 6f also presents the expression of the current flowing through the ZnO NWs resistance (*I*_1_ = *I*_ZnO NWs_) as a function of the WB components, input voltage (*V*_AC_) and output voltage (*V*_BD_). This expression was used to calculate the response of ZnO NWs to different light illuminations.

#### UV photodetector characterization

**Fig. 7:**
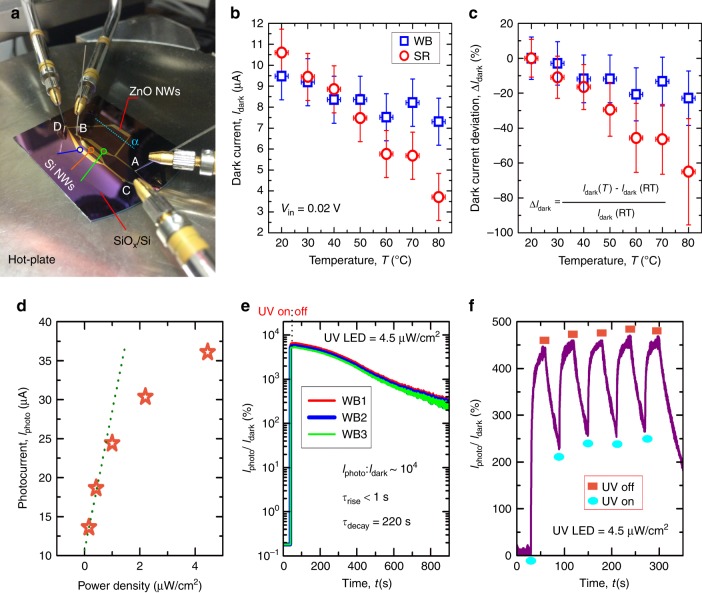
Photoresponse of Wheastone Bridge UV Photodetectors. **a** Photographic picture of Wheatstone bridge (WB) UV PD based on contact-printed ZnO and Si NWs; blue dotted line (α) shows the direction and place of the cut carried out to fabricate a UV PD based on a single resistance (SR). **b** *I*_dark_ and **c** Δ*I*_dark_ vs. *T* for WB and SR UV PDs. **d** ZnO NW current vs. UV LED power density, measured for 5 s under UV illumination, with a distance between NWs and UV LED of 5 cm, and using a *V*_in_ of 0.05 V. *I*_photo_*/I*_dark_ (**e**) single cycle and **f** multi-cycles measured over time and using a UV LED power density of 4.5 μW/cm^2^ and a *V*_*i*n_ of 0.05 V, keeping a distance between UV LED and the PD surface of 5 cm

For the sake of comparison, we have characterized the UV response of both UV PDs based on balanced WB and a single resistance (SR)^33^. Firstly, we have characterized up to 3 WB UV PDs using a Probe Station (Fig. 7) and a Semiconductor Device Analyser (Keysight B1500A). The probe station is provided with a temperature control, allowing us to carry out the electrical characterization of the PDs as a function of the temperature (*T*) ranging from room temperature (RT) to 80 °C. After the characterization of the WB UV PDs, these devices were cut (see α cutting line in Fig. 7a), insulating the array of electrodes with ZnO NWs, and resulting in UV PDs based on SR. Then, the same characterization was repeated on UV PDs based on SR. Figure 7b presents dark current (*I*_dark_) of WB and SR UV PDs, measured at *V*_in_ of 0.02 V, as a function of *T*. From that figure, one can deduce that *I*_dark_ decreases with *T* independently of the PD type, which can be explained due to a higher reactivity of oxygen species, leading to an increase of the oxygen absorbed along the ZnO NW surface, and therefore an increase of the surface-trapped charge density^33^. However, the decrease rate is observed to be higher in SR than in WB configuration (Fig. 7b). For *T* > 50 °C, the deviation of *I*_dark_ with respect to RT values (Δ*I*_dark_) is more evident in SR UV PDs than in WB (Fig. 7c), exhibiting a high Δ*I*_dark_ of 65% at *T* = 80 °C (at *T* = 80 °C, WB shows Δ*I*_dark_ ~ 20%). In addition, WB UV PDs show a constant Δ*I*_dark_ for *T* > 60 °C, in contrast to SR UV PDs that exhibit a continuous increasing trend with *T*. These results highlight one of the benefits of using WB circuits for sensing applications, which is to reduce the noise from changes in temperature. The WB is a good arrangement to demonstrate the applications possible with the developed contact-printing system, e.g., the fabrication of complex configurations based on different semiconductor NWs acting as sensing and electronic layers.

Error bars plotted in Fig. 7b, c correspond to five different devices fabricated and tested under the same experimental conditions described above. The variation observed in the *I*_dark_ measured at a specific temperature is associated to the characteristic conductivity of multi-NW-based devices. Considering the simplistic example where a single NW bridges a pair of conductive electrodes, the resulting device characteristics are relatively independent on the NW alignment with respect to the electrodes (see FIG. S9(a) and FIG. S9(b)). On the other hand, in multi-NW-based devices like the one we are presenting in this work, well-aligned arrays of NWs (see FIG. S9(c)) and quasi-aligned NWs bridging a pair of electrodes (see FIG. S9(d)), are expected to present the same characteristic conductivity. In this regard, we believe that further optimization of the NW alignment may not improve the crystallinity behaviour of the channel, and therefore the reproducibility and reliability of the resulting devices. However, in the scenario where NWs form lateral contacts the electric transport through grain boundaries (anisotropic conductivity) is expected and as a result the behaviour could resemble with a device made from polycrystalline materials^46^. Although anisotropic conductivity has been demonstrated in highly ordered assembled of NWs^47^, contact-printing technique still needs further investigations and improvements in order to achieve compact self-assembled monolayers of NWs, allowing the observation of anisotropic conductivity (see Table 1).

FIG. S9(d) describes the real situation observed in this work (Fig. 6(e1, e2)), where NWs are quasi-aligned, but they do not form any lateral junction, hindering contact/tunnelling conductivity between NWs. In this scenario, we believe that the devices fabricated in this work are dominated by the conductivity through the longitudinal axis of the NW. The multi-NW-based structure makes the resulting device characteristics reliable, i.e., the contact-printing procedure developed in this work is able to produce devices exhibiting similar characteristics as demonstrated in Fig. 7e.

Finally, the photoresponse and response time, including rise time (*τ*_rise_) and decay time (*τ*_decay_) of UV PDs in WB configuration, was observed as a function of the UV power density to evaluate the utility of our contact-printing system for fabrication of functional NW based devices. For that, we have used a UV light-emitting diode (LED) (365 < *λ* < 370 nm and an optical power of 200 mW at 700 mA) from RS Components (S5050) as UV light source to irradiate NW-based PDs. The power density of this UV LED has been calibrated by using a Si photodiode (BPW21 from Osram) as a function of the LED driving current and distance between the LED and the photodiode (see FIG. S10). For the characterization presented in Fig. 7, we have positioned the UV LED on top of the PD surface separated by a vertical distance of around 5 cm. Figure 7d presents the dependence of *I*_photo_ (measured at *V*_in_ = 0.05 V) and the power density of the UV source, exposing the PD surface for 5 s. From this figure, one can conclude that at lower illumination power densities (<1 μW/cm^2^) the PD exhibits a linear response which is consistent with the mechanism governed by the charge carrier photogeneration^48^. On the other hand, for higher illumination power densities (>1 μW/cm^2^), *I*_photo_ changes to a sublinear dependence which can be understood as a lack of hole-traps present at the NW surface, which drastically reduces the photogeneration mechanisms and lead to a saturation of the PD response.

The *I*_photo_/*I*_dark_ ratio has been analysed by exposing the ZnO NWs area of the PD to a UV light with a power density of 4.5 μW/cm^2^ for 30 s, while applying to the WB a *V*_in_ of 0.05 V (Fig. 7e). Results demonstrate a high performance of the fabricated UV PDs, obtaining a *I*_photo_/*I*_dark_ of around 10^4^ in three different devices (Fig. 7e), confirming the high sensitivity of NW PDs and validating the reproducibility and reliability of the developed contact-printing system (Fig. 1) and fabrication procedure (Fig. 6). Comparing the *I*_photo_/*I*_dark_ ratio obtained from a SR UV PD based on a single NW^33^ and the WB UV PD based on multiple NWs obtained in this work, the former shows a wide variation of *I*_photo_/*I*_dark_ ratios ranged between 10^2^ and 10^6^, depending on the NW diameter. In contrast, multiple NWs WB UV PDs with a distribution of NW diameters result in a similar *I*_photo_/*I*_dark_ ratio along three characterized devices (Fig. 7e).

We have also characterized the response time of the UV PDs under single- (Fig. 7e) and multi-cycles (Fig. 7f) of UV illumination. The best fit to data obtained by a double-exponential rise and decay functions results in a weight-averaged rise (*τ*_rise_) and decay (*τ*_decay_) time constants below 1 s and around 220 s, respectively. These values are comparable to those reported elsewhere for SR-based ZnO NWs UV PDs^33^. The long *τ*_decay_ obtained in our devices is in good agreement with those typically observed in oxide semiconductor-based PDs, demonstrating the existence of a persistent photoconductive effect in the multi-NW-based PDs^49,50^, making the PD conductivity to remain high after its illumination.

The similar response of three WB PDs confirms a better reliability of contact-printed multi-NWs compared to single NW-based UV PDs. It is clear that the use of multi-NWs is critical for uniform response and so is the use of method to print the NWs. Further, the durability of UV PDs characteristics has been evaluated over time and under multi-cyclic UV illuminations, exhibiting a stable performance (*I*_dark_, *I*_photo_/*I*_dark_, *τ*_rise_, *τ*_decay_) during cyclic test (Fig. 7f) and over several months of characterization.

#### Flexible UV photodetectors

**Fig. 8:**
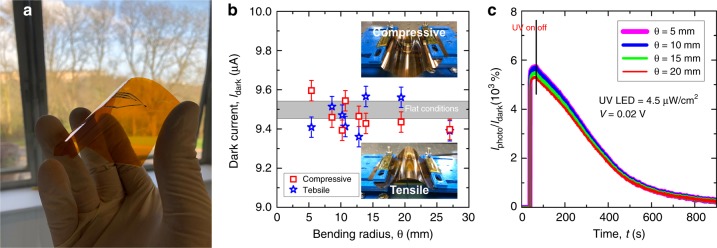
Characteristics of UV Photodetectors under Bending Conditions. **a** Photograph of the WB UV PD based on Si/ZnO NWs fabricated on a flexible PI substrate. **b** Dynamic bending characterization of flexible WB UV PDs, comprising compressive (top inset) and tensile bending (bottom inset) of the device and at different bending radii. **c** *I*_photo_/*I*_dark_ of WB UV PDs vs *θ* (in tensile conditions)

The validity of the developed contact-printing system to integrate different semiconductor NWs on rigid and flexible substrates has also been demonstrated here. Prior to the fabrication, a polyimide (PI) film (50 μm thick, from RS components) was attached to a Si(100) carrier wafer with a tape. Following the fabrication steps described in Fig. 6, Si and ZnO NWs were firstly contact-printed at specific areas over a PI substrate surface, and then Ti(4 nm)/Au(200 nm) electrodes were deposited by e-beam and pre-defined by photolithography and lift-off. After the fabrication of WB, the tape was dissolved in warm acetone (50 °C) to release the flexible WB UV PD from the carrier wafer (Fig. 8a). As-fabricated PDs exhibited characteristics similar to the rigid devices, i.e., *I*_dark_ ~μA (Figs. 7b and 8b) and *I*_photo_/*I*_dark_ ~ 10^4^ (Figs. 7e and 8c). To demonstrate the robustness of the resulting device, dynamic bending measurements were carried out at different bending angles (θ) ranged between 5 and 27 mm, and under both tensile and compressive conditions (Fig. 8b). The outcome from the bending experiments points out the robustness and stability of the fabricated PDs under tensile/compressive conditions and high θ, exhibiting a constant *I*_dark_ independently on the bending conditions (Fig. 8b). Furthermore, *I*_photo_/*I*_dark_ of the flexible WB UV PDs has been also obtained (using UV power density of 4.5 μW/cm^2^ and applying a voltage of 0.02 V) under different bending conditions, presenting a stable characteristic under θ (tensile conditions) ranged between 5 and 27 mm (Fig. 8c).

## Conclusions

This work shows the successful heterogeneous integration of ZnO and Si NWs on both rigid and flexible substrates, and over large areas through a home-made contact-printing system. The developed system has a close-loop configuration and allows us to carry out the transfer of both bottom-up (ZnO) and top-down (Si) NWs from growth substrate to a foreign substrate, achieving (1) high transfer-yields, i.e. preserving the as-grown NW length of 10 μm, NW crystalline structure, and NW morphology, (2) high NW densities (7 and 3 NWs/μm for ZnO and Si NWs), (3) low NW-to-NW spacings (ZnO NWs: 165 nm; Si NWs: 455 nm), (4) NW integration over areas from few mm^2^ to tens of cm^2^ and (5) NW integration of both Si/SiO_2_ rigid substrate and polyimide flexible substrate. Contact pressure has been theoretically and experimentally analysed to determine the optimum value for each kind of NW sample. For a donor substrate consisting of ZnO NWs vertically aligned on the surface of a Si substrate, we have observed that a contact pressure of around 50 kPa maximizes the NW density in the printed electronic layer. In addition, the system allows us to print NWs in a pre-defined area and by controlling the print area it is possible to tune the electronic properties (e.g., resistance) of the electronic layer. Accordingly, we have demonstrated the successful fabrication of a UV PD in a WB configuration, with ZnO and Si NWs as the resistive elements of the branches of WB. The characterization of the resulting UV PDs exhibits an excellent response to UV illumination (*I*_photo_/*I*_dark_ above 10^4^) and a relatively better stability to thermal effects—thanks to the WB self-compensation mechanism. The contact-printing system developed in this work has demonstrated high reproducibility and realibity, and therefore is a promising technology for the heterogeneous integration of different kinds of semiconductor and metal NWs, presenting opportunities for scalability over large areas and possibly the 3D integration on flexible substrates.

## Materials and methods

### Bottom-up ZnO NW synthesis

In the CVT process, ZnO micro-powder (<5 μm particle size, 3 N) is used as Zn source; that ZnO powder is mixed with graphite (C) powder (<20 μm particle size) resulting in a mixture with a 1:1 ZnO:C mass ratio. Then, the mixture and substrate are loaded in a ceramic crucible, which is transferred to the centre of the quartz tube. The sample is loaded on a quartz platform that rises its position with respect to the powder level, preserving the direct transport of powder from the source to the substrate. The CVT process is carried out at a temperature of 950 °C, leading to the carbothermal reduction of ZnO powder, producing Zn(g) and oxygen species to the ambient as described by:^38,39,51^

3 $${\mathrm{ZnO(s)}} + {\mathrm{C(S)}}\mathop{\longrightarrow}\limits^{{950^\circ {\mathrm{C}}}}{\mathrm{Zn(g)}} + {\mathrm{CO(g)}}$$

4 $${\mathrm{ZnO(s)}} + {\mathrm{CO(S)}}\mathop{\longrightarrow}\limits^{{950^\circ {\mathrm{C}}}}{\mathrm{Zn(g)}} + {\mathrm{CO}}_2{\mathrm{(g)}}.$$

### Top-down Si NW synthesis

Firstly, a SAM of sub-micrometric SiO_2_ SPs was dip-coated on Si(100) substrates (Fig. 2h), using operation parameters optimized elsewhere^52^. Thereafter, a reactive ion etching (RIE) process, using a CHF_3_/Ar gas flux of 25 sccm/18 sccm, 200 Watt, 30 mT and RT, was carried out for 10 min in order to shrink the size of the SPs, increasing the SP-to-SP spacing (Fig. 2i). Then, a 10–15 nm thick Ag film was thermally evaporated on top of the sample (Fig. 2j) and analysed by SEM (Fig. 2(j1)), followed by a sonication process for 5 min to remove the SPs (Fig. 2k), resulting in a metallic nano-mesh consisting of nano-pores as demonstrated by SEM (Fig. 2(k1)). Finally, MACE synthesis was carried out by dipping the sample in HF + H_2_O_2_ solution (Fig. 2l).

### Contact-printing system description

The vertical displacement of the donor substrate can be controlled with a minimum step size of 1 μm with a maximum load up to 10 N through a VT-21 linear stage motor (from Micronix USA). The load cell (Model 1004 from Vishay) has a rated output of 0.9 mV/V, a maximum rated capacity of 6 N and a maximum excitation voltage of 10 V, i.e., the voltage-to-force conversion factor (exciting the load cell at 10 V) is around 0.67 N/mV. The alignment between donor and receiver substrates is analysed by a Digital Microscope 1.3M (from RS Components). Once both donor and receiver substrate are in contact and the required applied pressure is reached, the receiver substrate sliding is carried out using a horizontal linear stage motor from Motorlink.

### Bending characterization

The performance of WB UV PDs fabricated on flexible PI substrates (Fig. 8a) has been studied under dynamic bending conditions, with both compressive (see bottom inset in Fig. 8) and tensile stress (see top inset in Fig. 8b). For this study, flexible WB UV PDs were mounted on a custom-made bending system, consisting of two linear stages VT-21L (from Micronix USA)—controlled by a Pollux Box (from PI MiCos) through a Labview software—and 3D printed platforms (insets of Fig. 8). The bending system allows an accurate control on the speed and position of the linear stages, resulting in a soft and precise bending of the sample under test. In this work, we have studied the response of flexible PDs to UV light, under bending radii ranged between 5 and 27 mm. For the sake of reproducibility, we have measured parameters such as *I*_dark_ and *I*_photo_/*I*_dark_, in static bending conditions, i.e., keeping the bending radius constant during the characterization. The area of the device covered by NWs, i.e., the sensing area of the PD shown in Fig. 6(e_1_) (ZnO NWs) and Fig. 6(e_2_) (Si NWs), have been placed in the centre of the curvature radius during the bending measurements, enhancing the reliability of the measurements.

## Electronic supplementary material

Movie 1

Movie 2

Supplemental Material File #1
