# Supplementary material for: Heterogeneous integration of contact-printed semiconductor nanowires for high-performance devices on large areas
Source: Microsyst Nanoeng. 2018 Aug 13;4:22. doi: 10.1038/s41378-018-0021-6 (PMC6220160; doi:10.1038/s41378-018-0021-6)
Supplement: Supplementary file 3 — Supplemental Material File #1 [file 41378_2018_21_MOESM3_ESM.pdf]

Heterogeneous Integration of Contact-printed Semiconductor Nanowires for High Performance Devices on Large Areas

Carlos García Núñez, Fengyuan Liu, William Taube Navaraj, Adamos Christou, Dhayalan Shakthivel, Ravinder Dahiya

Bendable Electronics and Sensing Technologies (BEST) Group, School of Engineering, University of Glasgow, G12 8QQ Glasgow, United Kingdom

Supplementary Information

Table of Figures

FIG. S1. Load Cell Calibration Curves

FIG. S2. Analysis of Donor/Receiver Substrates Alignment

FIG. S3. Contact-printing Software: Labview Interface

FIG. S4. Au NPs on Si(111)

FIG. S5. ZnO NW Morphological Characterization vs Ar Flow Conditions

FIG. S6. ZnO NWs Grown under Low Ar flow Conditions

FIG. S7. Towards Large-area Integration of NWs: Contact-printing Scalability

FIG. S8. Wheatstone Bridge Theory

FIG. S9. Conductivity Mechanism in Single and Multi-NWs Devices

FIG. S10. UV LED Power Density

Supplementary Information

Figures

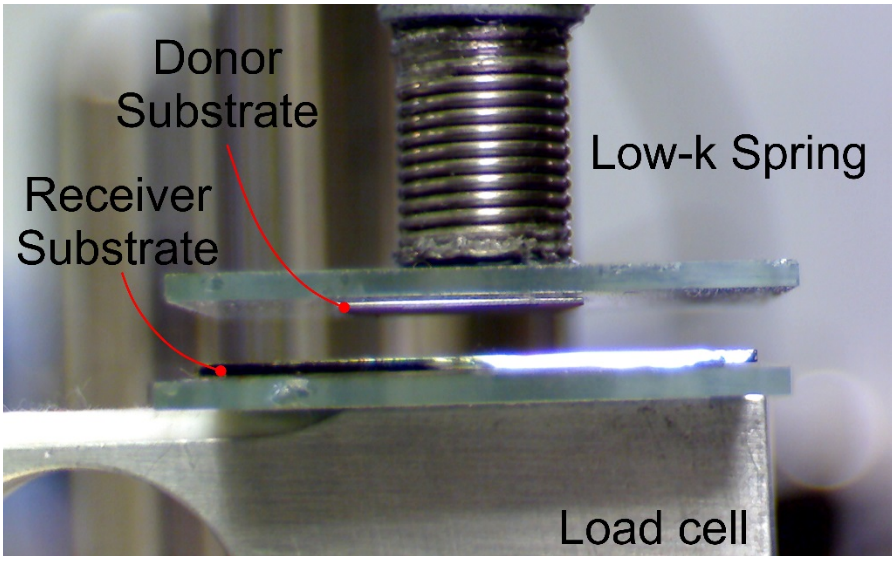

FIG. S1. Photographic picture of the donor/receiver substrate alignment taken by an optical microscope during the approaching step.

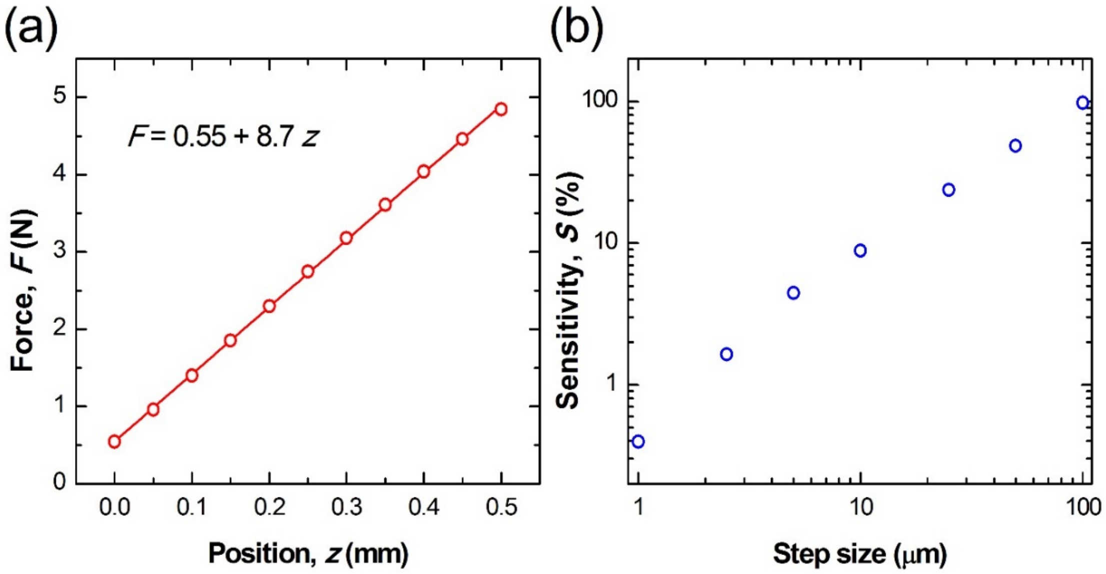

FIG. S2. (a) Force applied by the vertical motor vs. motor position relative to the contact position. (b) Load cell sensitivity as a function of the step size.

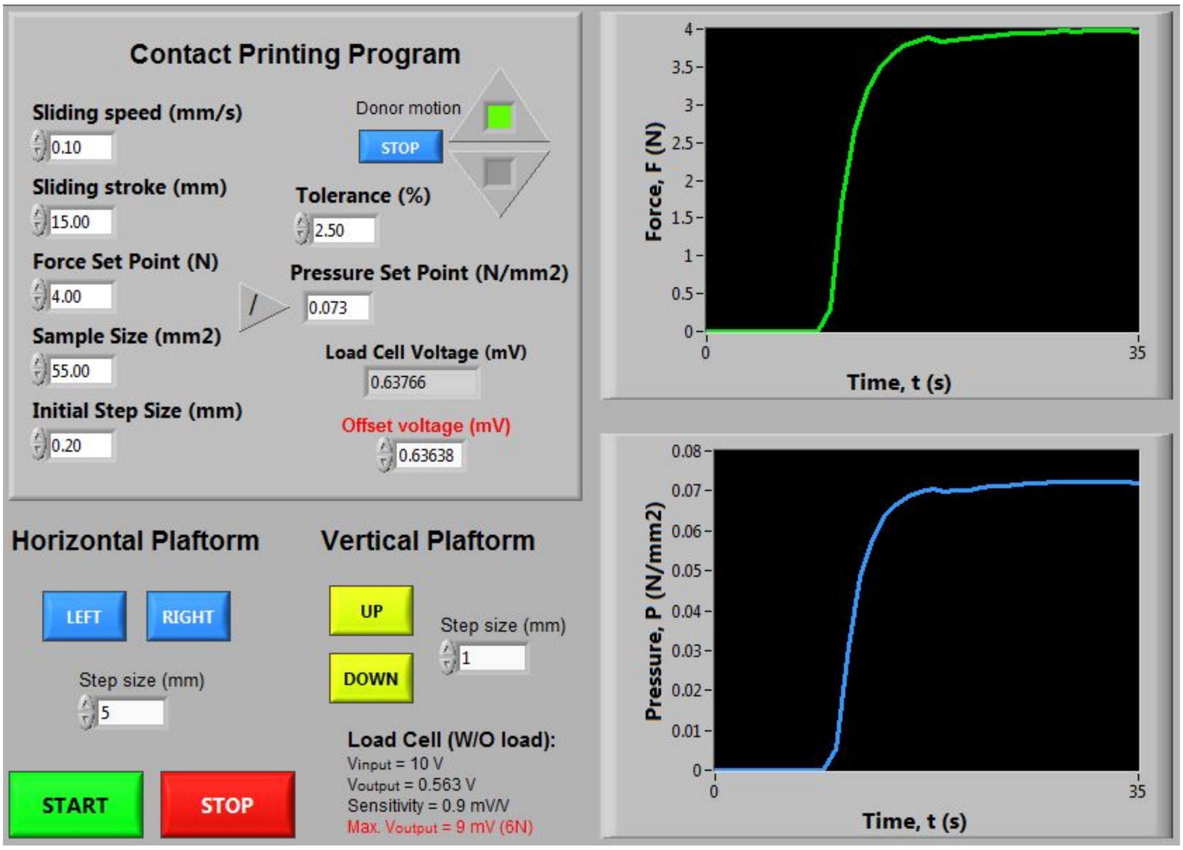

FIG. S3. Image of the Labview interface programmed to control and to monitor the contact-printing process. The program allows to set parameters such as sliding speed, sliding stroke, contact force, sample area, and step size of the vertical motor. In addition, the user can set the tolerance of the experiment which determines the accuracy of the reached contact pressure. Prior to the contact-printing process, the software asks the user to consider any offset measured by the load-cell, which has the role of "Tare" option available in every balance. At the bottom, the program allows the user to move vertical and horizontal linear stages and to adjust their step size. This is a useful feature not only to align donor and receiver substrates but also to print NWs at specific regions over the receiver substrate. On the right, one can find two plots, showing the contact force and pressure measured over time, permitting the user to monitor the correct functionality of the whole system.

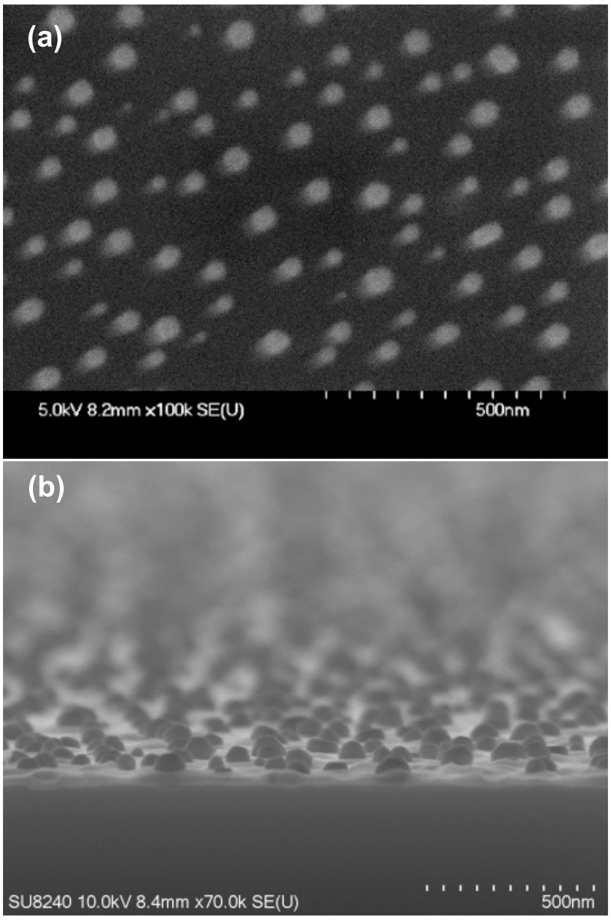

FIG. S4. (a) Top- and (b) cross-sectional views of Au NPs randomly distributed on Si(111) substrate obtained from the annealing of a 4-nm thick Au film at 1050°C for 10 min.

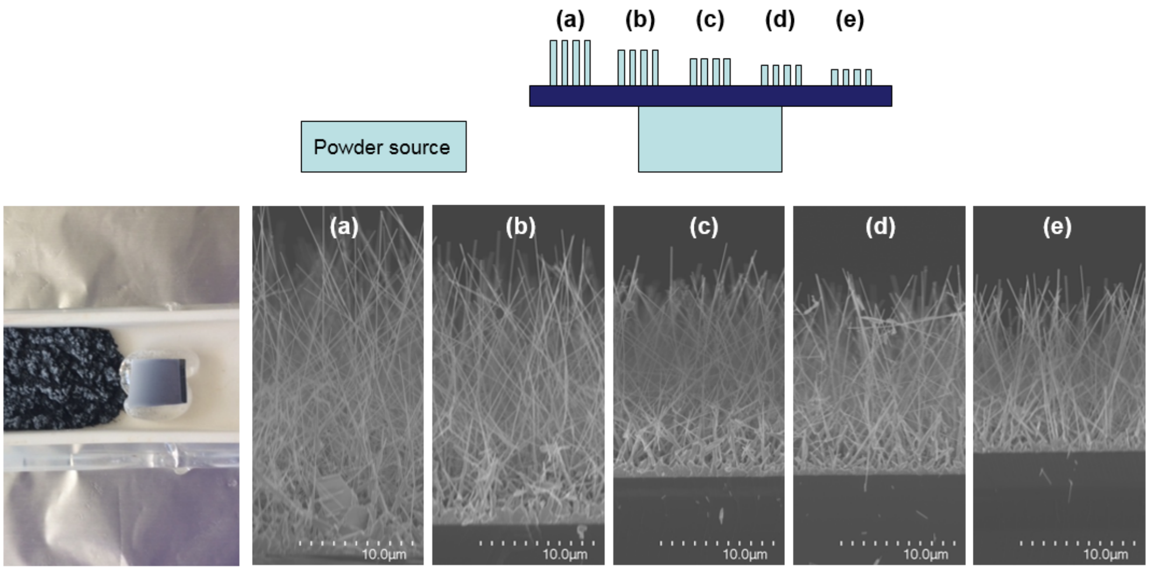

FIG. S5. (Left) Photographic picture of as-grown ZnO NWs, including the ZnO/C powder and the Si(111) substrate with ZnO NWs grown on top of its surface (whitish grey colour) using a low Ar flow of 500 sccm. (a-e) SEM images of ZnO NWs grown on a Si(111) substrate, analysed at different positions with respect to the Zn powder source, i.e. (a) and (e) being the closest and furthest analysed points along the substrate surface, respectively. From this SEM images one can conclude that the distance between the substrate and the powder source plays an important role on the morphology of the resulting NWs. Using Ar flows below 1000 sccm, we have observed a lack of NW length uniformity (a-e) mainly due to the variation of the gas species distribution along the substrate surface.

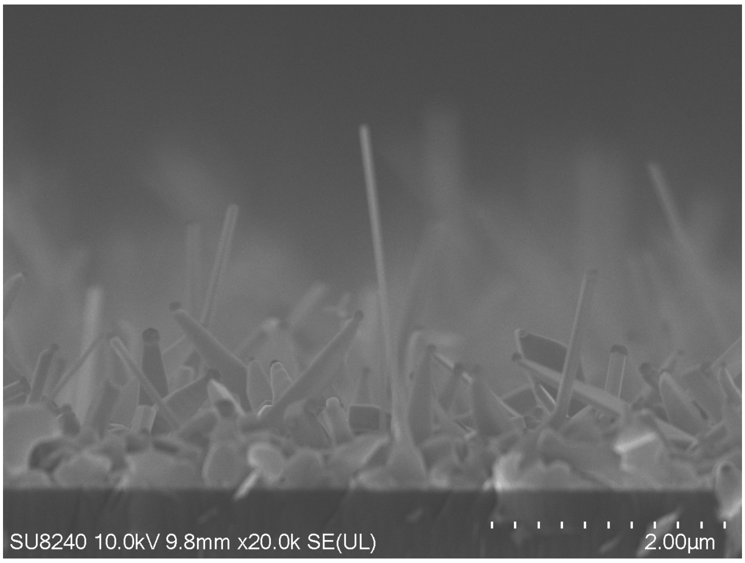

FIG. S6. SEM image of ZnO NWs grown on a Si(111) substrate using 250 sccm Ar flow. The use of an extremely low Ar flow strongly affects the morphology of the resulting ZnO NWs, which can even hinder the nucleation of ZnO with the shape of a NW, and promote the nucleation of other parasitic nanocrystals.

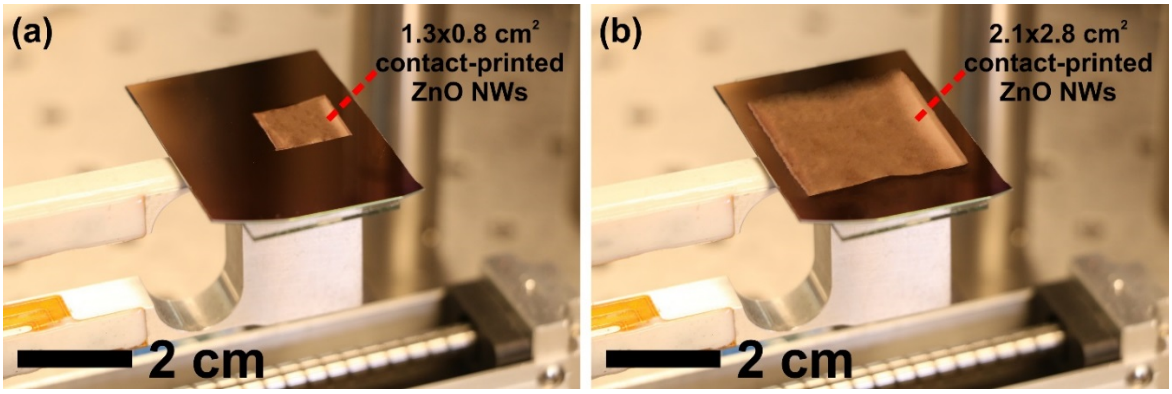

FIG. S7. Photographic pictures of the Si wafers right after the contact-printing of (a) 1.3×0.8 cm^2 and (b) 2.1×2.8 cm^2 ZnO NWs donor substrates. These pictures demonstrate the excellent scalability of the contact-printing system from few mm^2 towards tends of cm^2.

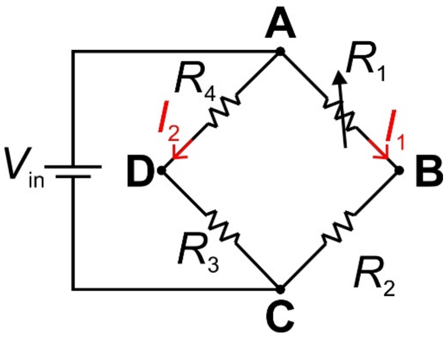

FIG S8. Wheatstone bridge circuit.

$V_{in} = V_A = i_1\,(R_1+R_2)$

$V_B = i_1\,R_2$

$V_A/V_B = (R_1+R_2)/R_2$

$V_B = [R_2/(R_1+R_2)]V_A$

$V_{in} = V_{AC} = i_2\,(R_3+R_4)$

$V_D = i_2\,R_3$

$V_A/V_D = (R_3+R_4)/R_3$

$V_D = [R_3/(R_3+R_4)]V_A$

$V_{out} = V_B - V_D = [R_2/(R_1+R_2) - R_3/(R_3+R_4)]V_{in}$

If WB is balanced $V_{out} = 0$, then:

$R_1 = R_2 = R_3 = R_4$

or

$R_1 / R_2 = R_4 / R_3$

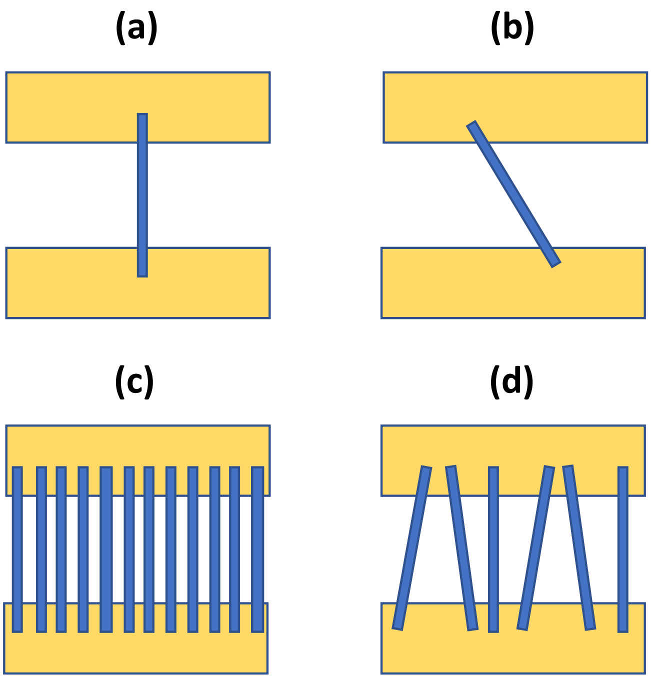

FIG. S9. 2D schematic diagram of (a,b) single and (c,d) multi-NWs bridging a pair of conductive electrodes. **Single NW based devices** could comprise (a) well-aligned or (b) randomly oriented NWs. **Multi-NW based devices** could comprise (c) well-aligned or (d) randomly aligned NWs.

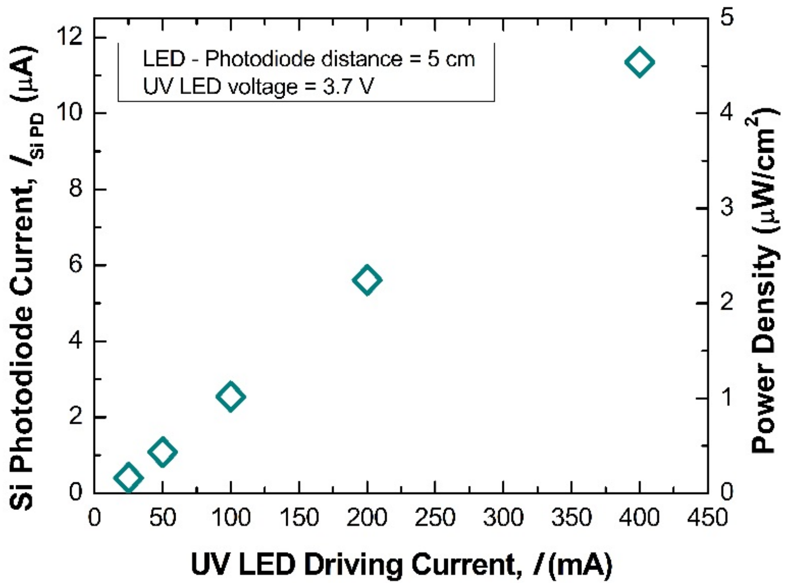

FIG. S10. Approximate power density of the UV LED light source vs. LED driving current (at 3.7 V) measured by a Si photodiode placed at 5 cm far from the LED surface. The photodiode current was transformed into a power by the sensitivity factor (S) of the device (S ~ 0.34 A/W) corrected by the wavelength (λ) factor of 0.3 (at λ = 390 nm), and the total area of illumination observed at aforementioned distance.
